# Insights into the mechanism of mycelium transformation of *Streptomyces toxytricini* into pellet

**DOI:** 10.1093/femsmc/xtad017

**Published:** 2023-08-21

**Authors:** Punit Kumar, Khushboo, Deepanshi Rajput, Kashyap K Dubey

**Affiliations:** Department of Morphology and Physiology, Karaganda Medical University, Karaganda 100008, Kazakhstan; Department of Biotechnology, Central University of Haryana, Mahendergarh 123031, India; Biomanufacturing and Process Development Laboratory, School of Biotechnology, Jawaharlal Nehru University, New Delhi 110067, India; Biomanufacturing and Process Development Laboratory, School of Biotechnology, Jawaharlal Nehru University, New Delhi 110067, India

**Keywords:** apical tip extension, hyphae branching, lipstatin, mass transfer limitations, microscopic analysis of pellet, pellet morphology, *Streptomyces*

## Abstract

Formation of the mycelial pellet in submerged cultivation of *Streptomycetes* is unwanted in industrial fermentation processes as it imposes mass transfer limitations, changes in the rheology of a medium, and affects the production of secondary metabolites. Though detailed information is not available about the factors involved in regulating mycelial morphology, it is studied that culture conditions and the genetic information of strain play a crucial role. Moreover, the proteomic study has revealed the involvement of low molecular weight proteins such as; DivIVA, FilP, ParA, Scy, and SsgA proteins in apical growth and branching of hyphae, which results in the establishment of the mycelial network. The present study proposes the mechanism of pellet formation of *Streptomyces toxytricini* (NRRL B-5426) with the help of microscopic and proteomic analysis. The microscopic analysis revealed that growing hyphae contain a bud-like structure behind the apical tip, which follows a certain organized path of growth and branching, which was further converted into the pellet when shake flask to the shake flask inoculation was performed. Proteomic analysis revealed the production of low molecular weight proteins ranging between 20 and 95 kDa, which are involved in apical growth and hyphae branching and can possibly participate in the regulation of pellet morphology.

## Introduction

*Streptomyces* (Gram-positive bacteria) is one of the most explored microorganisms at the commercial scale for synthesizing natural products. During submerged fermentation, filamentous microorganisms exhibit morphologies between diffused mycelia and pellets as per the culture conditions and type of microbial strain (Hobbs et al. 1989). *Streptomyces toxytricini* is recognized for producing lipase-inhibitory natural product lipstatin, used in an FDA-approved anti-obesity drug (Kumar and Dubey 2015, 2016). Like other species of the genus, *Streptomyces toxytricini* also exhibits pellet morphology in submerged fermentation (Kumar and Dubey 2017). Mycelial morphologies are associated with producing the desired natural product. Moreover, the pellet morphologies affect the rheology of a medium, heterogeneous mass transfer, and downstream processing (Rioseras et al. 2014, Wang et al. 2017). Therefore, regulation of pellet morphology is necessary for industrial processes.

Studies conducted at the molecular level suggested the involvement of many genes and proteins that control the mycelium transformation or morphological differentiation of *Streptomyces* (Vassallo et al. 2020, Wu et al. 2020). *Streptomyces’* growth occurs from the spore into vegetative hyphae, regulated by the AMP receptor protein Crp (Derouaux et al. 2004). Further growth of the hyphae takes place by tip extension and branching, where polarity protein DivIVA, the first molecular marker of hyphal tips, plays a key role (Flardh et al. 2012). It was suggested that during the growth of hyphae, the development of cross-walls also takes place, which protects hyphae from fission and forms a multinucleated compartment (Jakimowicz and van Wezel 2012). Moreover, many protein complexes of *Streptomyces* like Scy, Tat secretion system, SsgA, and CslA are also associated with the apical tip growth of *Streptomyces*. In *Streptomyces*, cellulose synthase-like proteins, which are responsible for the synthesis of beta-glucan-containing polysaccharides, play an essential role in tissue morphogenesis, hyphal tip growth, and morphological differentiation (Noens et al. 2007, Hempel et al. 2012, Willemse et al. 2012, Holmes et al. 2013). It was reported that SsgA is involved in identifying a location for developing the septum and germination site (Noens et al. 2007). Interestingly, Scy, ParA, and FilP proteins are assumed to interact with DivIVA to control the apical growth of mycelia (Ditkowski et al. 2013, Holmes et al. 2013). Scy is a scaffold protein that functions as an apical dominance regulator, while FilP is a cytoskeletal protein that affects the hyphal shape (Khushboo et al. 2023). One more protein HyaS has been found conserved in streptomycetes and associated with the regulation of pellet morphology by maintaining hyphal contacts (Koebsch et al. 2009).

Moreover, it was also suggested that some extracellular materials (proteins, sugars, and DNA) work as adhesives and are involved in forming pellet-like structures and providing protection. Extracellular DNA, hyaluronic acid, teichoic acids, and CslA are associated with *Streptomyces*’ pellet morphology (Kim and Kim 2004, Ultee et al. 2020). It was found that cellulose synthase-like protein CslA was also identified near the hyphal tip, which was involved in growth and structural transformation. It was also studied that it interacts with DivIVA and regulates biopolymer formation with glycosidic bonds near hyphal tips. The physical phenomenon, such as oxygen transfer and shear rate, also influence the morphology of *Streptomyces* (Ribeiro et al. 2021). Directive and quick-acting approaches, like the addition of microparticles and macroparticles, have also been used to regulate mycelial morphology (Böl et al. 2020, Yue et al. 2021).

In the previous report, authors reported that culture conditions (pH of culture medium, medium composition, agitation rate, and inoculum size) influence the pellet size of *S. toxytricini* (Kumar and Dubey 2017). Still, the mechanism of mycelial transformation into a pellet has not been elaborated. Though pellet size was reduced with changes in culture conditions, biomass formation was also reduced. Thus, the mechanism of pellet formation must be studied to reduce the pellet size or maintain dispersed mycelial morphology without affecting the biomass. In the present study, authors attempted microscopic analysis of hyphae growth and branching, which further transformed into the pellet. Additionally, a partial analysis of proteins (produced by bacteria in pellet and culture medium) was performed to understand the role of proteins in pellet formation.

## Materials and methods

### Microorganism and growth conditions

*Streptomyces toxytricini* NRRL B-5426 strain was obtained from the Agricultural Research Service (NRRL), Department of Agriculture, USA. The bacterium was grown in a shake flask as per the conditions mentioned in Kumar and Dubey (2017). The incubation temperature was maintained at 27.5°C till the appearance of visible colonies. Well-grown colonies of *S. toxytricini* were pink in color, elevated, circular in shape, and had a specific odor.

### Analysis of pellet morphology

For pellet morphology analysis, an inoculum of *S. toxytricini* was prepared by transferring a loopful of bacteria (*S. toxytricini*) from a Petri plate into a shake flask, modified from Kumar and Dubey (2017).

After incubation, pellets were settled down and centrifuged at 5000 rpm for 10 min to remove the culture medium. Aggregated pellets were washed thrice with 0.1 M sodium phosphate buffer of pH 7.2 ± 0.1 and stored for further processing. The Gram staining and methylene blue staining of pellets were performed to analyze morphology and shape. Visualization was done in a compound microscope. For scanning electron microscopy (SEM) of pellets, primary fixation of pellets was performed in 2.5% glutaraldehyde and 2% paraformaldehyde in 0.1 M sodium phosphate buffer at pH 7.2 ± 0.1. Further dehydration, fixation and coating, and SEM analysis were done at Advanced Instrumentation Research Facility, Jawaharlal Nehru University, New Delhi (India). For biomass analysis, pellets were kept for drying at 50°C in a hot air oven till the appearance of constant weight.

### Proteomic analysis of samples

Intracellular and extracellular proteins produced by *S. toxytricini* during growth were extracted separately from the growth medium and pellets. The acetone precipitation method was used for protein extraction from culture broth, and for protein isolation from pellets, pellets were processed in lysis buffer at pH 7.0 (Hobbs et al. 1989, van Veluw et al. 2012). Isolated proteins were analyzed by 10% SDS PAGE.

## Results and discussion

### Growth of *S. toxytricini*

The growth of *S. toxytricini* in submerged fermentation showed that if inoculum was transferred from the Petri plate to the shake flask, it produced diffused mycelia and pellets of smaller size, while the transfer of inoculum from the shake flask to the shake flask produced visible pellets in the culture broth. Thus it can be assumed that in the Petri plate, bacterial mycelium was not programmed for pellet formation, but in the culture broth, *S. toxytricini* produces some chemical compound (intracellular or extracellular) that directs the formation of pellets at the submerged level. Microscopic examination of *S. toxytricini*, which was grown using inoculum from a Petri plate, revealed that mycelial aggregation took place, but the majority of these aggregations were clumps, loose mycelia, and smaller pellets (up to 20 µm). While shake flask to shake flask inoculation produced pellets of larger size (30 µm–2 mm) as major morphological form and very less loose mycelia. Thus, it may be assumed that culture conditions influence the transformation of mycelia into pellets, and when *S. toxytricini* is grown in submerged conditions, it produces some biochemicals or proteins that control pellet formation. According to a previously reported study, this heterogeneity of mycelial forms (loose, clump, and pellet) is maintained in a broth medium (Kumar and Dubey 2017). Similarly, some studies have reported that variation in culture conditions directly affected the pellet morphology of bacterial strains, but such mycelial morphology is unwanted for industrial processes (Flardh et al. 2012, van Veluw et al. 2012, Rioseras et al. 2014).

### Morphology of pellets

**Figure 1.**
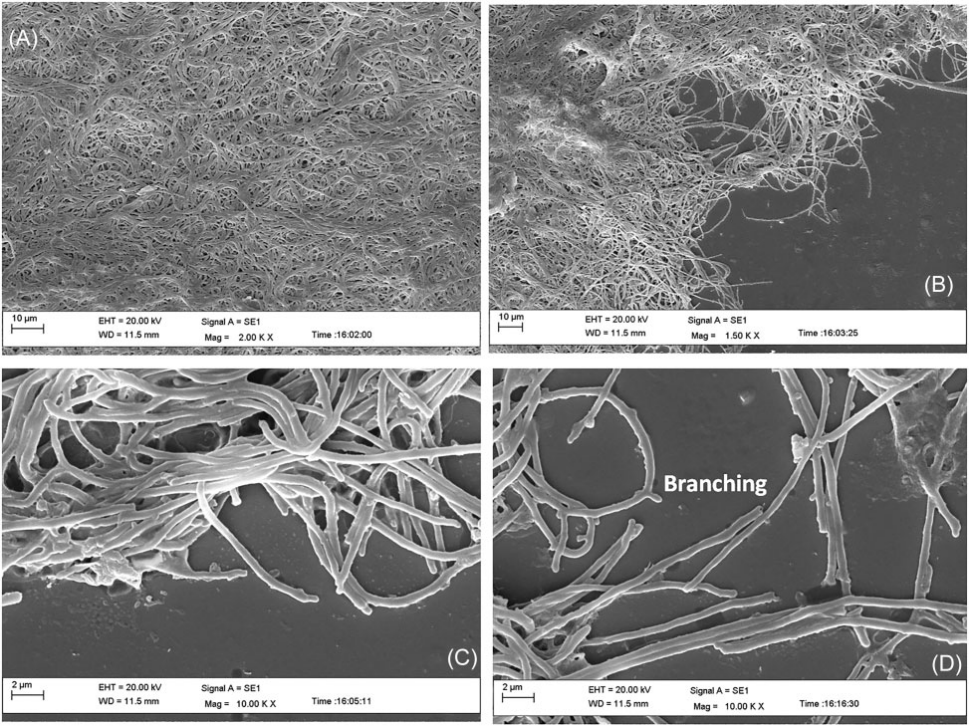
Analysis of pellet morphology and mycelial structure by SEM. **(A)** Morphology of pellet at 10 µM scale. **(B)** Surface of pellet showing free mycelia, which show growth and branching at 10 µM scale. **(C)** Structure of hyphae showing association with each other and branching at growing tips (2 µM scale). **(D)** Apical growth and branching of hyphae (2 µM scale).

For a better insight into pellet structure, the morphology of pellets was analyzed by scanning electron microscope (Fig. 1). In this study, it was observed that pellet formation takes place by interwoven hyphae (Fig. 1A), where hyphae are joined with each other and form a compact network (Kim and Kim 2004, Koebsch et al. 2009). The surface of the pellet has loose mycelia, which are sites of growth, and these mycelia undergo fragmentation during agitation to form a new pellet (Fig. 1B) (Koebsch et al. 2009). Further magnification (2 µm scale) revealed that hyphae are attached to each other and form tight junctions (Fig. 1C). Microscopic analysis revealed that growing hyphae bear bud-like structures behind the apical tip, and these bud-like structures form branches that were developed at regular intervals (Fig. 1D) (Hempel et al. 2012). Thus it may be assumed that branching behind the apical tip and tight junctions assist in compact pellet formation. As reported earlier, such types of association protect hyphae from fission and produce multi-nucleoid structures (Jakimowicz and van Wezel 2012). The microscopic analysis strongly supported the observations of previous researchers, such as; the generation of multiple polarity centers (Holmes et al. 2013), growth and development of pellet by tip extension, branching, and cross-wall formation (Flardh et al. 2012, Hempel et al. 2012, Khushboo and Dubey 2023).

### Proteomics analysis

**Figure 2.**
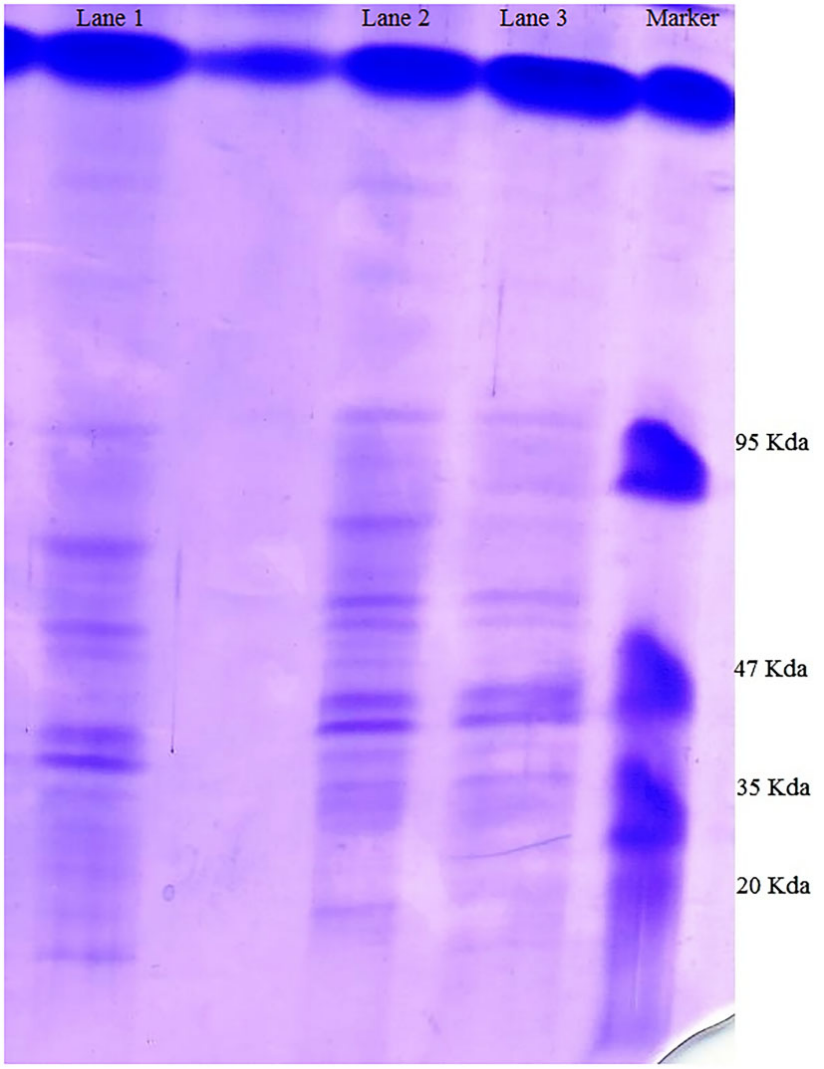
Analysis of extracellular and intracellular proteins of *S. toxytricini* by SDS-PAGE. Lane 1 represents total intracellular proteins, and lanes 2 and 3 represent total extracellular proteins isolated from the culture medium. The marker lane is a molecular-weight marker.

Researchers have reported many proteins (DivIVA, Scy, FilP, SsgA, ParA, Tat, CslA, Afsk, CRP, HyaS, TrpM, and Mat complex, etc.) involved with apical growth, formation of septa, branching in growing hyphae (Noens et al. 2007, Hempel et al. 2012, Willemse et al. 2012, Ditkowski et al. 2013, Holmes et al. 2013, van Dissel et al. 2018, Vassallo et al. 2020, Zhang et al. 2023). To further elucidate the involvement of different proteins behind the pellet formation of *S. toxytricini*, secreted proteins in the culture medium and intracellular proteins were analyzed by electrophoresis. In this study, the partial analysis of proteins (intracellular and extracellular) was conducted. It was observed that large numbers of proteins of different molecular weights were produced at the intracellular and extracellular levels ranging from 95  to <20 kDa (Fig. 2). It is assumed that many of these proteins are involved in pellet formation. It was observed in Fig. 1D that growing hyphae form branches behind the apical tip. Studies have reported the expression of DivIVA (21.5 kDA) at the growing tip, which is suggested to interact with Scy, ParA, and FilP to regulate pellet formation (Ditkowski et al. 2013, Holmes et al. 2013). It has been suggested that Scy regulates a number of polarity centers by associating with DivIVA for new tip construction during branching (Holmes et al. 2013). Including this, Scy was found to sequester DivIVA and initiate the formation of new polarity centers (Holmes et al. 2013). The protein Scy is also assumed to associate with ParA to hyphal tips and control polymerization of ParA. The Scy-ParA association is assumed to be involved in the transition of hyphal elongation into sporulation (Ditkowski et al. 2013). Thus these proteins need further analysis for their involvement in pellet formation. Including this, the presence of other proteins involved in the growth and branching of hyphae into pellet formation needs to be analyzed before making any final conclusion.

### Mechanism of pellet formation

**Figure 3.**
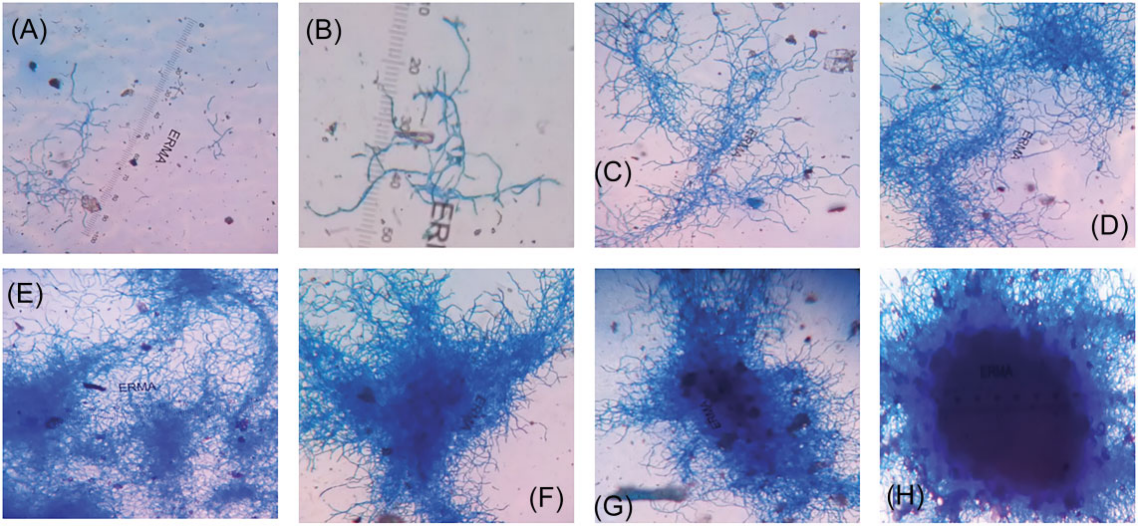
Proposed mechanism for pellet formation. **(A)** Diffused mycelia, **(B)** mycelia are coming together, **(C)** mycelia are clumping, **(D)** mycelial clumps are more visible, and a reaction center-type structure is formed, which possibly directs pellet formation, **(E)**–**(G)** mycelial clumps are more condensed, and **(H)** pellet formed and periphery having mycelia.

In the previous study, the authors discussed the process of pellet formation (Kumar and Dubey 2017) (Fig. 3). DivIVA is found to be associated with cell wall synthesis, genetic competence, and chromosome segregation during sporulation (Labajova et al. 2021). It has also been suggested that DivIVA, with some other proteins (Scy, ParA, and FilP), regulates the formation of polarisome and forms branches in hyphae. Though continuous agitation of culture medium enables clumping of mycelia, some external biopolymers may assist the association of hyphae with each other, resulting in compact pellets. To analyze this process, microscopic observation was conducted to understand the pattern of pellet formation. It was revealed that hyphae perform growth and branching behind the apical tip during growth. The growing hyphae come in close proximity to form a network of hyphae, which forms a reaction center-like structure. This structure further grows and associates with other hyphae and finally converts into a compact structure (Fig. 3). This hypothesis is also supported by the study that apical growth regulates cell polarity, which determines the morphology of the pellet (Hempel et al. 2012, Holmes et al. 2013). Including this, researchers have demonstrated cross-wall formation during the growth of streptomycetes, which compact the structure into pellets (Flardh et al. 2012, Zhang et al. 2023). Thus it may be suggested that pellet formation involves the process of hyphae growth, development of polarity, branching, and formation of cross-walls, which results in the transformation of hyphae into compact pellets.

## Conclusions

Streptomycetes are filamentous microorganisms reported for producing many valuable natural products, including antibiotics and enzymes. The present study has led to the following conclusions:

1. The hyphae of these microbes represent a distinct morphological form, i.e. pellet during submerged cultivation and undesirable for industrial processes. Though studies have reported the production of antibiotics using pellet morphology. Investigators have reported the involvement of culture conditions and biomolecules controlling the morphology of hyphae.
2. In this study, microscopic analysis of morphological forms of *S. toxytricini* at different times revealed that during submerged growth, the growth of hyphae took place by tip extension, branching behind the apical tip.
3. The growing hyphae possibly attached to each other and formed cross-walls, which further transformed into compact pellets (Flardh et al. 2012, Hempel et al. 2012).
4. The partial proteomic analysis to understand the role of proteins in pellet formation revealed that there is the presence of low-weight proteins in the pellet and culture medium, which are possibly involved in pellet formation.

Still, a detailed study of proteins like DivIVA, Scy, FilP, SsgA, ParA, Tat, CslA, Afsk, CRP, HyaS, and Mat complex is required.
